# Cost-effectiveness analysis of metagenomic next-generation sequencing versus traditional bacterial cultures for postoperative central nervous system infections in critical care settings: a prospective pilot study

**DOI:** 10.3389/fcimb.2025.1710412

**Published:** 2025-10-28

**Authors:** Ying Tian, Ningyuan Xu, Yuqing Chen, Zimeng Xu, Jian-Xin Zhou, Linlin Zhang

**Affiliations:** ^1^ Department of Critical Care Medicine, Beijing Tiantan Hospital, Capital Medical University, Beijing, China; ^2^ School of Information Science and Technology, Beijing University of Technology, Beijing, China; ^3^Stanley and Karen Pigman College of Engineering, University of Kentucky, Lexington, KY, United States, Beijing, China; ^4^ Department of Critical Care Medicine, Beijing Anzhen Hospital, Capital Medical University, Beijing, China; ^5^ Beijing Shijitan Hospital, Capital Medical University, Beijing, China

**Keywords:** central nervous system infections (CNSIs), metagenomic next-generation sequencing(mNGS), pathogen culture, cost-effectiveness analysis, incremental cost-effectiveness ratio(ICER)

## Abstract

**Background:**

Early and accurate pathogen identification is crucial for managing central nervous system infections (CNSIs). While Metagenomic Next-Generation Sequencing (mNGS) offers rapid and sensitive pathogen detection, its cost-effectiveness in postoperative neurosurgical patients in critical care settings remains underexplored. Our study aims to investigate the clinical health economic value of mNGS in detecting pathogens of CNSIs after neurosurgery.

**Methods:**

In this prospective pilot study, 60 patients with CNSIs at Beijing Tiantan Hospital ICU (March 2023-January 2024) were randomized 1:1 to mNGS or conventional pathogen culture groups. A decision-tree model compared cost-effectiveness using incremental cost-effectiveness ratios (ICERs). A decision-tree model was used to compare the cost-effectiveness between mNGS and traditional pathogen culture methods using incremental cost-effectiveness ratios (ICERs).

**Results:**

From March 2023 to January 2024, 60 patients were included. mNGS demonstrated superior diagnostic efficiency with shorter turnaround time (1 vs 5 days; _P_<0.001) and lower anti-infective costs (¥18,000 vs ¥23,000; _P_=0.02). Despite higher detection costs (¥4,000 vs ¥2,000; _P_<0.001), the ICER of ¥36,700 per additional timely diagnosis suggested cost-effectiveness at China’s GDP-based WTP threshold. No significant differences in hospitalization duration or total costs were observed (_P_>0.05).

**Conclusion:**

mNGS improves diagnostic efficiency and reduces antimicrobial expenditure for postoperative CNSIs in critical care, demonstrating favorable cost-effectiveness when considering clinical outcome gains.

## 1 Introduction

Central nervous system infections represent a critical neurological emergency characterized by mortality rates exceeding 10-30% (Wong et al., 2024). The acute progression of CNSIs necessitates rapid pathogen identification to guide targeted antimicrobial therapy, as empirical regimens based on regional antibiotic resistance patterns often lead to delayed appropriate treatment—a key risk factor for poor outcomes (Reynolds, 2024). Current diagnostic paradigms combine traditional pathogen culture with emerging metagenomic next-generation sequencing (mNGS) technologies (Brouwer and van de Beek, 2017; Piantadosi et al., 2021). While culture remains the clinical gold standard, its limitations are well-documented: cerebrospinal fluid cultures demonstrate 5%-10% sensitivity in post-neurosurgical infections, with time-to-result averaging 5–7 days. In contrast, mNGS achieves 85-92% sensitivity within 24 hours, enabling earlier therapeutic optimization (Benoit et al., 2024; Tian et al., 2024; Wang X. et al., 2024). However, this diagnostic advantage comes at substantial cost—mNGS expenses typically exceed conventional methods by 10 to 20 times (Liao et al., 2023), raising critical health economic questions about its value proposition in resource-constrained settings.

Despite growing clinical adoption, evidence gaps persist regarding the comprehensive impact of mNGS-guided management. Existing studies predominantly focus on diagnostic accuracy metrics (Howell et al., 2018), neglecting clinical outcomes, economic consequences and strategic value.

This study addresses these gaps through a comparative health economic evaluation of 60 post-neurosurgical CNSI patients. Utilizing a decision-tree model with probabilistic sensitivity analysis, we quantify the incremental cost-effectiveness ratio (ICER) of mNGS versus culture-based diagnosis, contextualized against China’s 2023 GDP per capita (¥89,000). Our findings provide urgently needed evidence for optimizing diagnostic protocols in neurosurgical intensive care, balancing clinical urgency with fiscal responsibility.

## 2 Materials and methods

### 2.1 Study design and participants

This is a single-center randomized controlled trial conducted in the Department of Critical Care Medicine of Beijing Tiantan Hospital affiliated with Capital Medical University, with the main subjects being patients with a comprehensive clinical diagnosis of CNSIs after neurosurgery. Sixty patients with a comprehensive clinical diagnosis of CNSIs after neurosurgery were randomly assigned 1:1 to two groups (mNGS group and control group). Patients in the experimental group were diagnosed by cerebrospinal fluid pathogen culture and mNGS, while patients in the control group only received a diagnosis by pathogen culture.

This study included patients with CNSIs who were clinically diagnosed after neurosurgery in the Department of Intensive Care Medicine, Beijing Tiantan Hospital, affiliated with Capital Medical University. Exclude patients with unqualified samples and patients who refuse to sign informed consent. To ensure the study’s quality and the results’ reliability, we assembled a team of clinicians, microbiologists, bioinformatics analysts, and two laboratory technicians to supervise and guide the study process.

This study was reviewed and approved by the Ethics Committee of Beijing Tiantan Hospital Affiliated to Capital Medical University (Approval ID: KY2023-018-02), and the informed consent ID was 20221120.

### 2.2 Diagnostic process

From the patients’ perspective, we established a decision analysis model to accurately diagnose CNSIs after neurosurgery. The model considered two detection strategies, mainly mNGS and pathogen culture. The patients were randomly divided into the mNGS group and the control group (it should be noted that after the two groups of patients were clinically diagnosed with CNSIs, they could be immediately given empirical treatment without waiting for the results of mNGS or pathogen culture).

**Figure 1:**
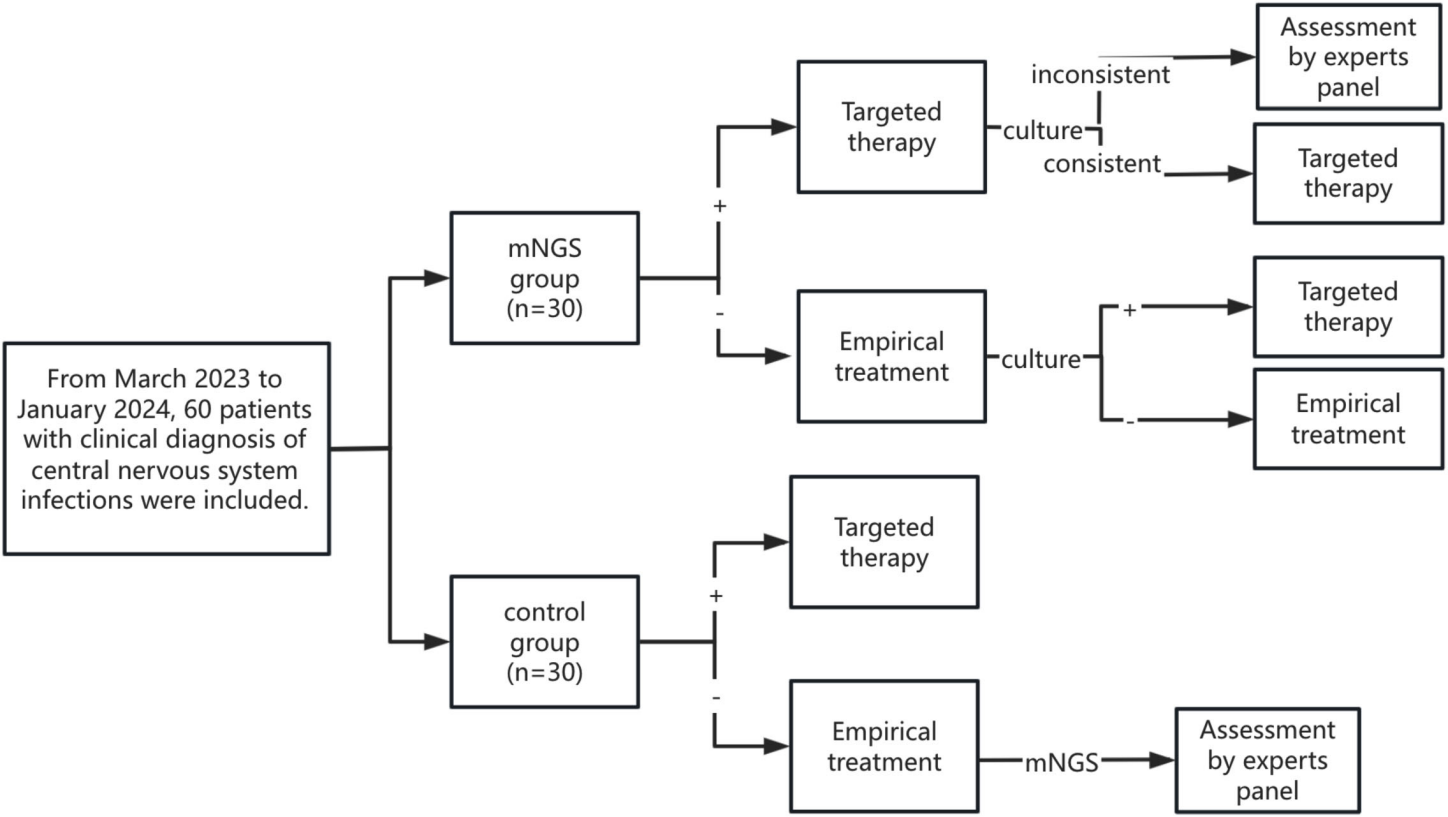
Flow chart.

As shown in **Figure 1**, patients in the mNGS group underwent both CSF mNGS and pathogen culture, and mNGS results were usually earlier than pathogen culture results. Therefore, patients in the mNGS group first reported the pathogen status based on mNGS and expert panel opinion and adjusted or continued the current treatment regimen. Subsequently, if the CSF pathogen culture results were consistent with the mNGS results, the patient’s current treatment regimen was continued. If the CSF pathogen culture results were inconsistent with the mNGS results, the expert panel discussed and adjusted or continued the current treatment regimen. When no pathogenic bacteria were detected in mNGS, empirical therapy was continued, and the treatment regimen was adjusted according to the pathogen culture results.

After a clinical diagnosis of CNSIs, only CSF was collected for pathogen culture in the control group, and mNGS was not performed. The treatment regimen was adjusted according to the results of the pathogen culture. If the patient’s culture results were negative and there was no improvement from empirical treatment, the patient’s CSF was collected for mNGS. The patient’s treatment regimen was adjusted based on mNGS results and expert panel evaluation.

### 2.3 Cost-effectiveness analysis

We constructed a Markov decision tree (TreeAge Pro 2022) comparing cost components, and effectiveness metrics. The cost-effectiveness evaluation employed the incremental cost-effectiveness ratio (ICER) as the primary metric. China’s GDP-based willingness-to-pay (WTP) threshold was set at 1–3 times the 2023 per capita GDP (¥89,000) following WHO recommendations.

The incremental-cost-effectiveness ratio (ICER) represents the incremental cost of each additional quality adjusted life year (QALY) received by the intervention compared to the control program. The calculation formula is ICER = Δc/Δe = (C2-C1)/(E2-E1). In general, when the threshold of ICER is set to be less than or equal to a per capita GDP, it is considered that the intervention program has health economic benefits and is worthy of recommendation. When ICER is between one and three times per capita GDP, it is considered acceptable to implement an intervention plan. Still, when ICER is greater than three times per capita GDP, it is considered that the intervention plan has no health economic benefits.

### 2.4 Quality-adjusted life years

Since QALYs could not be calculated for this study population, ΔE was defined as the incremental treatment response score for CNSIs at patient discharge. The scoring criteria were as follows:

Score 0: Baseline clinical status of CNSIs at enrollment.

Score 1: Infection controlled at discharge, but cerebrospinal fluid or other infection indicators not normalized, requiring continued anti-infective treatment.

Score 2: CNSIs cured at discharge, cerebrospinal fluid indicators normalized, no further anti-infective treatment needed.

Score -1: Infection not effectively controlled at discharge, condition worsened compared to enrollment, but patient requested discharge against medical advice.

Score -2: Patient died, with CNSIs not controlled at time of death.

### 2.5 Statistical analysis

Measurement data were expressed as mean and standard deviation. The independent sample t-test statistically analyzed data conforming to a normal distribution. Data that did not conform to the normal distribution were statistically analyzed using the Mann-Whitney test. Categorical variables were expressed as counts and percentages and analyzed using χ2 tests. All analyses used GraphPad Prism 10 with P<0.05 considered significant.

## 3 Results

### 3.1 Patient characteristics

The study enrolled 60 post-neurosurgical patients with clinically confirmed CNSIs between March 2023 and January 2024, randomized equally to mNGS (n=30) and traditional culture (n=30) groups. Baseline demographics was shown in **Table 1**. In the mNGS group, there were 23 male and 7 female patients, while in the control group, there were 17 male and 13 female patients. The median age of patients in the mNGS group was 42 years, compared with 47.5 years in the control group. In terms of primary diseases, the proportion of patients with craniocerebral tumors was the most significant, 66.7% (20/30) in the mNGS group and 76.7% (24/30) in the control group. Cerebrovascular disease ranked second in both groups, with 13.3% (4/30) in both mNGS and control groups.

**Table 1:** Patient characteristics.

	mNGS group (n=30)	Control group (n=30)
Gender		
Male	23 (76.7%)	17 (56.7%)
Female	7 (23.3%)	13 (43.3%)
Age	42 (31.75,55.75)	47.5 (34.75,55)
Primary disease		
Brain trauma	3 (10.0%)	1 (3.3%)
Brain tumor	20 (66.7%)	23 (76.7%)
Cerebrovascular disease	4 (13.3%)	4 (13.3%)
Other	3 (10.0%)	2 (6.7%)
Time cost (Days)		
Length of hospital stay	26.5 (19.25,36.25)	26.5 (19,34.75)
Length of ICU stay	17 (14,25.75)	17.5 (11.25,28)
Length of resistance to infection	12 (11.25,18.25)	16.5 (11,22)
Length of pathogen detection time	1 (1,1)	5 (5,6)
Hospitalization cost (ten thousand yuan)	19.0 (14.7,24.0)	16.8 (13.7,23.5)
Antibiotic costs (ten thousand yuan)	1.8 (1.3,2.6)	2.3 (1.8,4.7)
Testing costs (ten thousand yuan)	0.4 (0.35,0.7)	0.2 (0.13,0.67)

### 3.2 Cost-effectiveness analysis

We also measured the cost of hospitalization and infection-related costs in both groups, as well as length of hospital stay, length of stay in ICU, duration of anti-infective treatment, and duration of testing. The median length of hospital stay was 26.5 days in both groups. The median length of hospitalization was 17 days in the mNGS group and 17.6 days in the control group. The median duration of anti-infective treatment was 12 days in the mNGS group and 16.5 days in the control group. The mNGS detection is usually obtained on the morning of the second inspection day, so the pathogen detection time of the mNGS group is uniformly 1 day, and less than 24 hours is also recorded as 1 day. The median time of pathogen culture in the control group was 5 days. The median total hospitalization cost was ¥190,000 in the mNGS group and ¥168,000 in the control group. The median cost of antibiotics for CNSIs in the two groups was 18,000 yuan and 23,000 yuan, respectively. The CSF laboratory examination related to CNSIs (mainly including routine cerebrospinal fluid, cerebrospinal fluid biochemistry, cerebrospinal fluid culture, lumbar puncture, lumbar cisterna drainage, etc.) The mNGS test is the test cost, where the cost of sending a single sample of metagenomic second-generation sequencing test is 3000 yuan (RMB). Therefore, in terms of test cost, the median cost of patients in the mNGS group was 4,000 yuan, and that of patients in the control group was 2,000 yuan. Significant differences were found in pathogen detection time, antibiotic costs, and testing costs between the mNGS and control groups.

As shown in **Table 2**, 20 patients in the control group were cured, 3 were improved, 4 were aggravated, and 3 died upon discharge, with an average score of 1.1 ± 1.5. At discharge, 24 patients in the mNGS group were cured, 5 were improved, 1 was aggravated, and 0 died, with an average score of 1.7 ± 0.6.

**Table 2:** Infection treatment effect scores of patients in the two groups at discharge.

Scores	mNGS group	Control group
Cure (2)	24	20
Improve (1)	5	3
Aggravate (-1)	1	4
Death (-2)	0	3
Average	1.7 ± 0.6	1.1 ± 1.5

**Figure 2:**
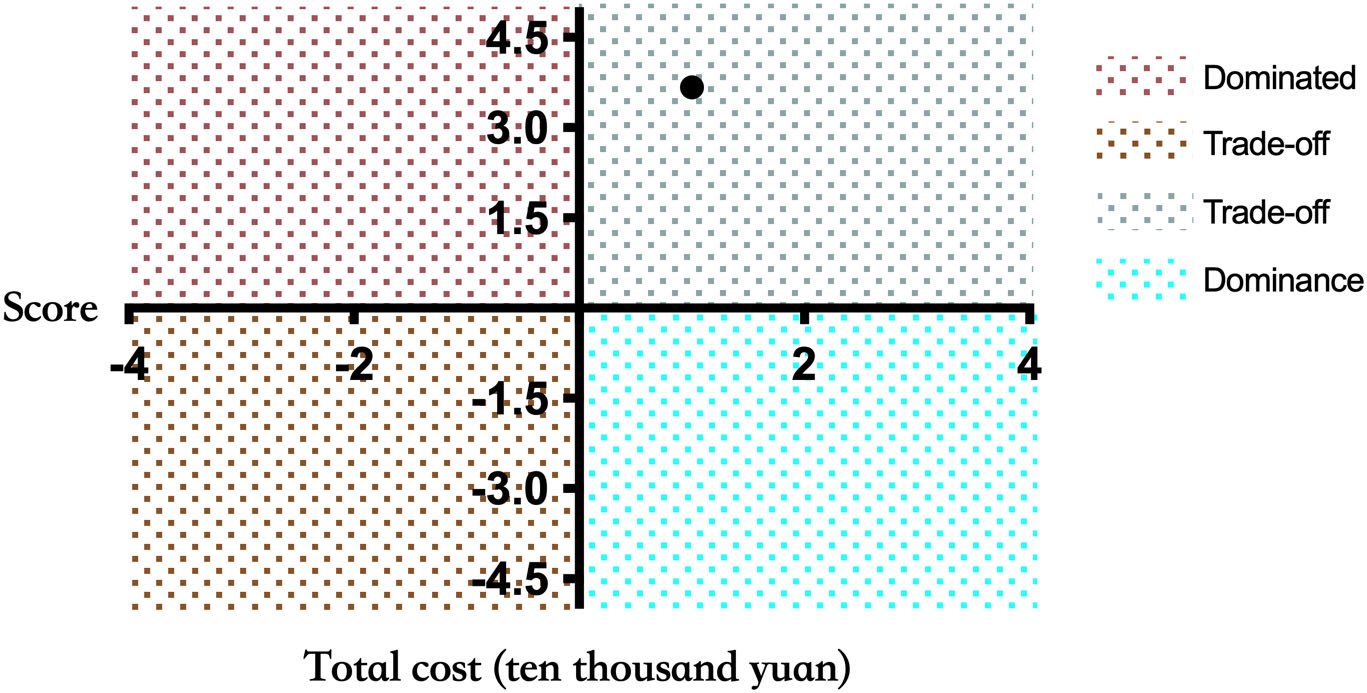
Incremental cost-effectiveness plan. The X-axis represents the patient’s treatment effect score, and the Y-axis represents the total cost of the patient during hospitalization (ten thousand yuan). If the results are in the fourth quadrant, the mNGS group is dominant compared to the control group. When the results are in the second quadrant, the cost of the mNGS group is higher and the effect is worse, which is Dominated. When the result is in the first or third quadrant, a threshold is needed to aid decision making.

Therefore, ICER (ten thousand yuan) = (19-16.8)/(1.7-1.1) = -1.2/0.6 = 3.67 can be calculated according to the formula. As shown in **Figure 2**, ICER value falls in the first quadrant in the incremental cost-effect plan, and it can be concluded that ICER < 1 per capita GDP (based on China’s per capita GDP in 2023:89,400 yuan/person), and the mNGS strategy demonstrates health economic significance.

### 3.3 Length of hospital stay and admission to intensive care unit

**Figure 3:**
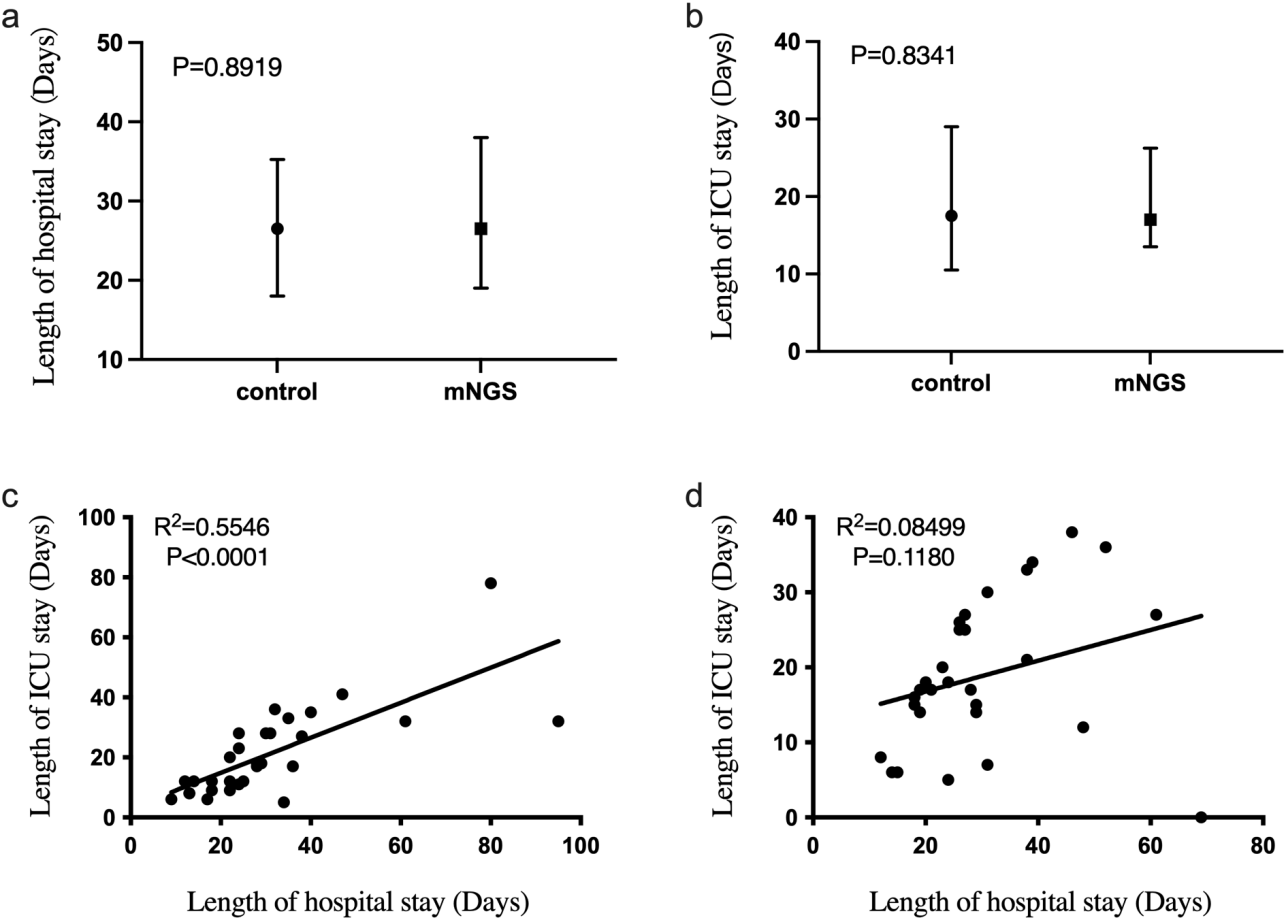
The difference of hospitalization time and ICU admission time between mNGS group and control group. **(a)** Comparison of total hospital stay between mNGS group and control group, *P* > 0.05; **(b)** The hospitalization time of mNGS group and control group was compared, *P* > 0.05; **(c)** Linear regression of total hospital stay and ICU stay in control group, *R2* = 0.5546, *P* < 0.0001; **(d)** Linear regression of total hospital stay and ICU stay in mNGS group, *R2* = 0.08499, *P* > 0.05.

The length of stay in the intensive care unit also directly affects the patient cost. Therefore, we compared the length of hospital and intensive care unit stays of patients in the mNGS and the control groups. As shown in **Figure 3**, 3a was the total length of hospital stay (median (interquartile)) of the two groups, and 3b was the length of admission to the ICU (median (interquartile)) of the two groups. There is no significant difference between the length of hospital stay (P = 0.8919) and the length of stay in the ICU (P = 0.8341) of the two groups. Linear regression analysis showed a significant correlation between ICU stay and total hospital stay in the control group (R² = 0.5546, P < 0.0001, **Figure 3C**). However, in **Figure 3D**, the linear regression R2 = 0.08499 and P greater than 0.05 for the duration of hospitalization in the ICU and the total duration of hospitalization of patients in the mNGS group showed no significant correlation between them. We also added linear regression results between the length of ICU stay and hospital stay for all 60 patients included. Where R2 = 0.3581 and P value less than 0.0001 suggest that the length of stay in the ICU of patients in this study population was significantly correlated with the total length of hospital stay. However, the correlation R2 value was considerably lower than the control group’s (**Supplementary Figure 1**).

### 3.4 Anti-infective treatment time and detection time

**Figure 4:**
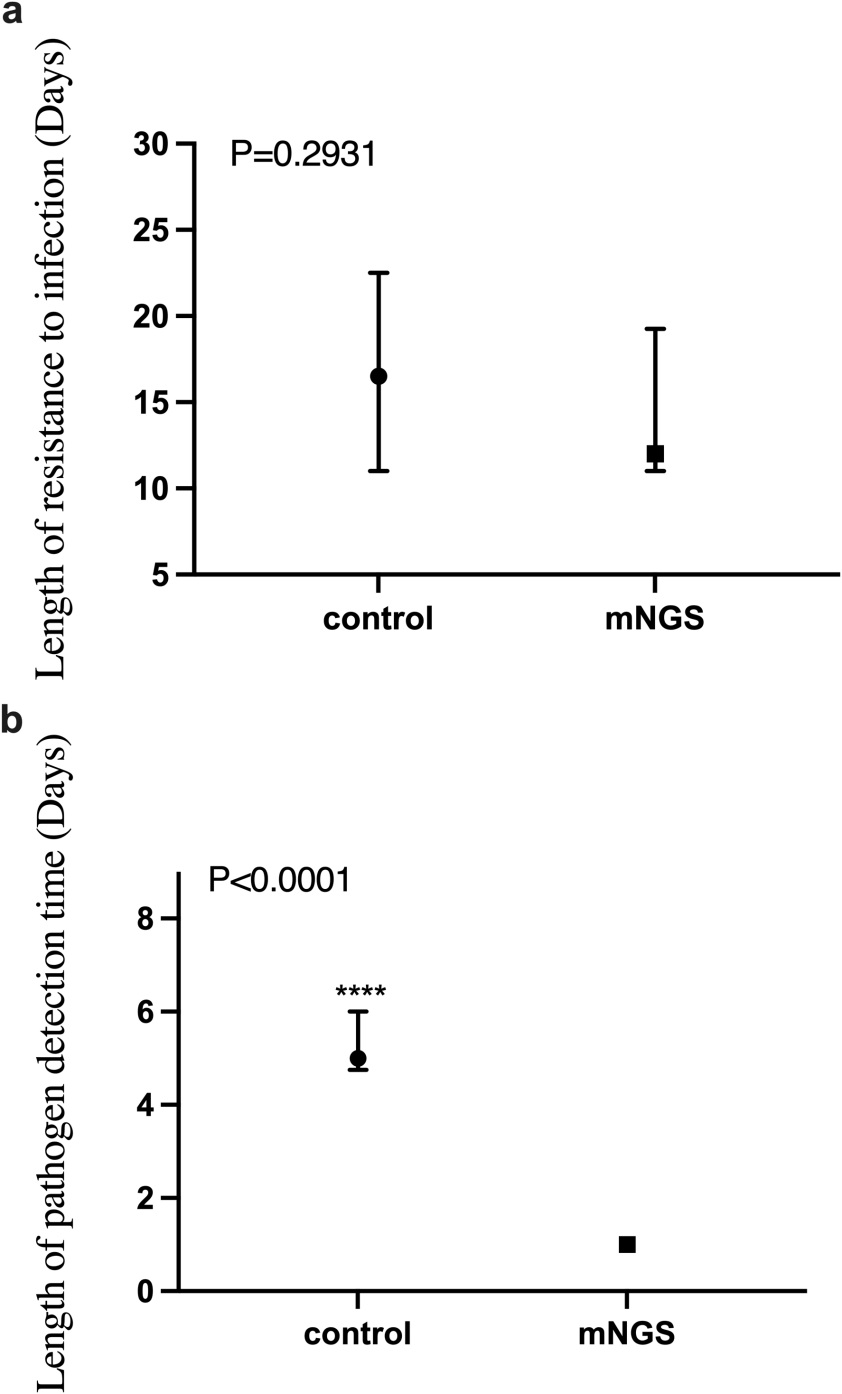
The difference of anti-infective treatment time and detection time between mNGS group and control group. **(a)** Comparison of anti-infection treatment time between mNGS group and control group, *P* > 0.05; **(b)** Comparison of pathogen detection time between mNGS group and control group, *P* < 0.0001. ****P ≤ 0.0001.

As shown in **Figure 4A**, there was no significant difference in the duration of anti-infective treatment between the mNGS group and the control group (P = 0.2931). This suggests that emerging diagnostic methods using mNGS technology do not significantly improve the duration of anti-infective treatment compared to traditional methods.

However, another advantage of mNGS technology is its rapid detection capability, often providing accurate pathogen detection results within 24 hours of submission. This is critical for the clinical management of infectious diseases. Our independent sample non-parametric test on the detection time of the two groups showed that the detection time of the mNGS group was significantly shorter than that of the control group (P < 0.0001), as shown in **Figure 4B**.

### 3.5 Hospitalization costs, anti-infection treatment costs and testing costs

**Figure 5:**
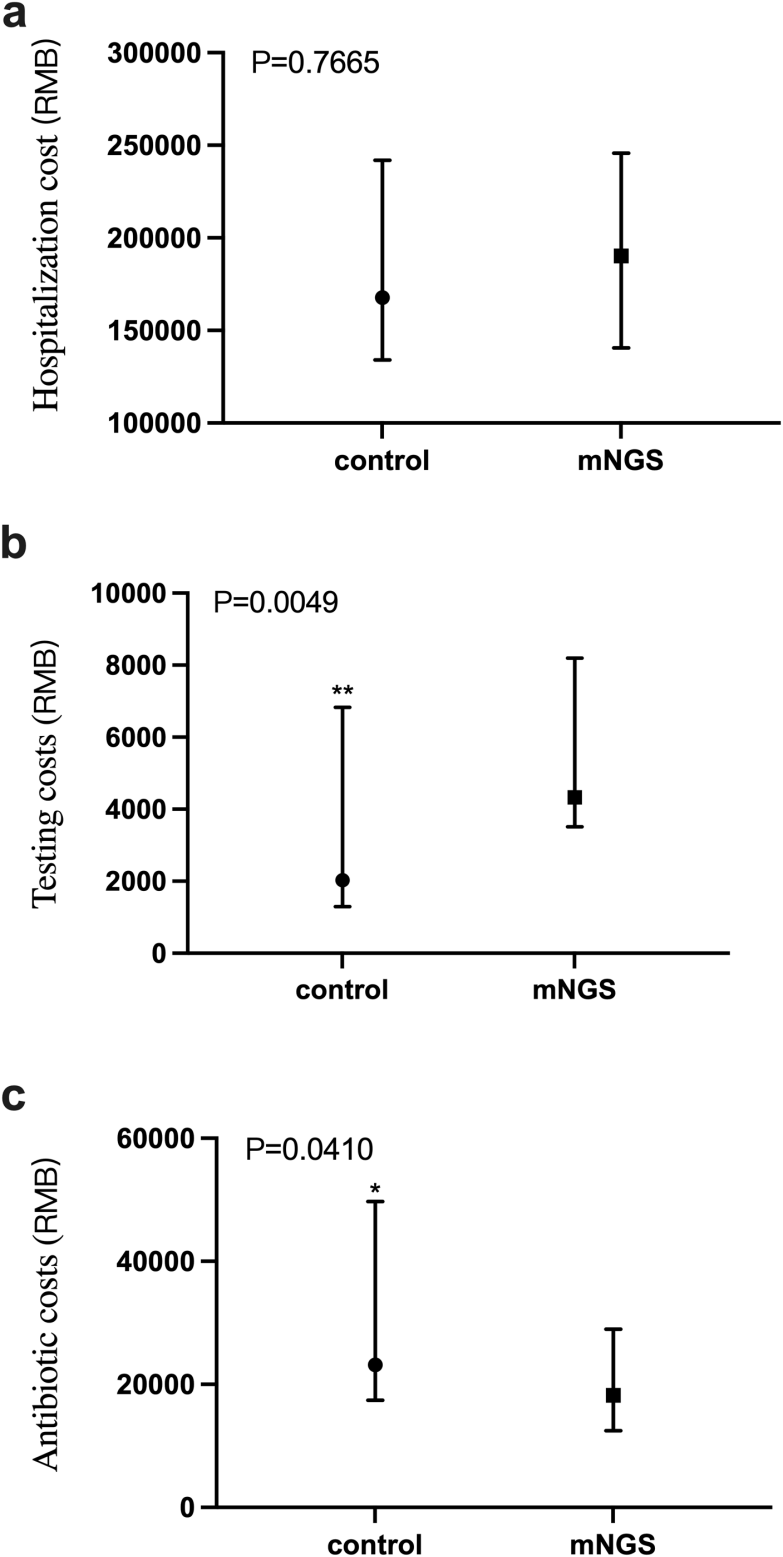
Comparison of total inpatient cost, anti-infective treatment cost and detection cost between mNGS group and control group. **(a)** Total inpatient cost in mNGS group and control group, *P* > 0.05; **(b)** The cost of pathogen detection in mNGS group and control group, *P* < 0.05; **(c)** The cost of anti-infection treatment in mNGS group and control group, *P* < 0.05. *P ≤ 0.05, **P ≤ 0.01.

The total cost of patient hospitalization is directly related to the incremental cost-effectiveness ratio. Here, we compare the total inpatient costs of the two groups. As shown in **Figure 5A**, the Mann-Whitney test was conducted on the total inpatient cost of patients in the mNGS group and the control group, and the P value was 0.7665, indicating that there was no significant difference between the total inpatient cost of patients in the two groups.

As shown in **Figure 5B**, the cost of pathogen detection for patients in the mNGS and the control groups was a P value of 0.0049, indicating a significant difference in the cost of detection between the two groups. The detection cost of the mNGS group was significantly higher than that of the control group. **Figure 5C** shows the cost of anti-infective treatment for patients in the mNGS group and the control group, with a P value of 0.0410, indicating a significant difference in the cost of anti-infective therapy between the two groups, in which the cost of anti-infective treatment in the mNGS group was significantly lower than that of the control group.

Linear regression analysis showed a significant correlation between anti-infective treatment cost and total hospitalization cost in both the control group (R² = 0.5750, P < 0.0001, **Supplementary Figure 2**) and the mNGS group (R² = 0.4832, P < 0.0001, **Supplementary Figure 2**), indicating that anti-infective treatment cost directly influences total hospitalization cost.

## 4 Discussion

CNSIs have become a global public health problem, characterized by high morbidity, high mortality, and high costs to the healthcare system. In recent years, the incidence of hospital-acquired infections has been on the rise, especially in intensive care units, where the incidence of Acinetobacter baumannii and Klebsiella pneumoniae have increased yearly (Liao et al., 2023; Wang Z. et al., 2024). Pathogen culture and mNGS techniques are commonly used to detect the etiology of CNSIs after neurosurgery (Brouwer and van de Beek, 2017). Our previous studies have shown that mNGS has advantages over culture-based methods in diagnosing CNSIs, but the detection cost is higher. There are limited systematic studies on the economic benefits of using these two different diagnostic strategies to treat patients with CNS infections. Therefore, this study aimed to evaluate the clinical benefit of two tests for patients diagnosing CNSIs.

Through the primary outcome of our study, it can be found that patients who adopted the precise diagnosis method of mNGS had a higher cure rate of CNSIs upon discharge and less aggravation of the disease. In contrast, the treatment of patients in the control group was slightly worse than that in the mNGS group. The calculated ICER value shows that the ICER value of the mNGS group falls in the first quadrant of the incremental cost-effectiveness plan, which indicates that the metagenomic second-generation sequencing method for the diagnosis of CNSIs can obtain better therapeutic effects within the acceptable cost and range. This means that mNGS is a diagnostic option with health and economic benefits. However, in this study, the study sample size and the characteristics of the study population may also affect the wide availability of the results. Given the potential advantages of metagenomic second-generation sequencing technology in diagnosis, therapeutic effect and economic benefits, the sample size can be expanded, and long-term follow-up observation can be conducted further to verify its impact in different populations and disease stages.

A study systematically reviewed the cost-effectiveness of second-generation sequencing versus traditional genetic disease diagnostic approaches. It explored the economic evaluation of second-generation sequencing in diagnosing genetic diseases (Howell et al., 2018). The study showed that the total cost of early targeted whole exome sequencing was lower than that of late targeted whole exome sequencing ($677,081 versus $738,136). In addition, early strategies using whole exome sequencing are cheaper and more effective than standard experiments. The cost of exome and gene sequencing is higher in the diagnostic pathway than in other genetic tests. However, the standard detection method has a high cumulative cost, long detection times and low efficiency. Exome sequencing as a secondary strategy is the least expensive (Rezapour et al., 2023). Because of its relatively high cost, second-generation sequencing is often performed as a last-resort diagnostic test. However, when second-generation sequencing is performed as a first-line test, the additional cost of testing avoided for confirmed patients may compensate for the high cost of the test (Dragojlovic et al., 2018). Studies have also investigated the cost-effectiveness of NGS and single gene testing for molecular diagnosis in patients with metastatic non-small cell lung cancer. The findings suggest that implementing second-generation sequencing technology in a Spanish reference center will be a more cost-effective strategy for molecular diagnosis of patients with metastatic non-small cell lung cancer than traditional single-gene testing. Cheng et al (Cheng et al., 2021). study assessing the diagnostic and economic value of second-generation sequencing technology and single gene testing in biomarker detection of metastatic non-small cell lung cancer. The report states that the implementation of second-generation sequencing technology is feasible and can be done at a reasonable cost, as second-generation sequencing technology is a multi-molecular diagnostic tool capable of overcoming the limitations of traditional sequencing technology. Current molecular diagnostics for advanced cancers allow for improved and economically sustainable molecular analysis. The study conducted by Weymann et al (Weymann et al., 2018). focused on economic assessments of second-generation sequencing technologies published between 2000 and 2016 and aimed to describe the availability and scope of economic evidence. They highlight the significant increase in economic evaluations of second-generation sequencing technologies published between 2014 and 2016 and the differences in cost-effectiveness outcomes between studies based on the methodology employed, the comparator chosen, and the source of funding.

In addition, we are also concerned about the use of antibiotics in patients with CNSIs. In recent years, antibiotic resistance has been a significant health and economic burden on healthcare systems worldwide, as well as on patients and their families (Carson and Weese, 2024; Omwenga and Awuor, 2024). In hospital settings, invasive procedures are often performed extensively because patients are clustered together. The use of antibiotics is very high in this situation (Golkar et al., 2014). According to the statistical results of our study, the cost of anti-infection treatment in the mNGS group was significantly lower than that in the control group (*P* < 0.05, with a significant difference). Applying the mNGS detection strategy has more health-economic benefits from the anti-infection treatment perspective than traditional microbial detection. mNGS technology was used to diagnose the etiology of central nervous system in early stage, and targeted therapy was adopted to reduce the multi-use of broad-spectrum antibiotics. In the long run, it could reduce the increase in antibiotic resistance.

Notably, while mNGS significantly reduced detection time and anti-infective drug costs, it did not significantly reduce hospital or ICU length of stay or total hospitalization costs in this study. The lack of correlation between ICU stay and total hospital stay in the mNGS group, unlike the control group, might suggest that other factors (e.g., primary disease severity, surgical complications) became more dominant drivers of length of stay when pathogen diagnosis was expedited. </mark> This warrants further investigation.

The significant reduction in anti-infective costs in the mNGS group aligns with the potential for early targeted therapy, minimizing broad-spectrum antibiotic use. This is crucial in the context of rising antimicrobial resistance.

This study has several limitations. First, its single-center nature and small sample size limit the generalizability of the findings. The generalizability of these findings may be limited by the single-center design and small sample size. Future multi-center studies with larger cohorts are needed to validate these results across diverse populations and healthcare settings. Second, the cost-effectiveness analysis relied on a self-defined effectiveness score rather than QALYs, which limits comparability with other health economic studies. Future research should incorporate standardized outcome measures. Third, we compared mNGS only to culture; other diagnostic methods like multiplex PCR were not evaluated and deserve attention in future studies. Furthermore, potential confounders such as variations in underlying disease severity and surgical procedures were not adjusted for in this preliminary analysis and should be considered in future larger studies. Despite these limitations, this pilot study provides valuable preliminary evidence supporting the clinical utility and cost-effectiveness of mNGS for post-neurosurgical CNSIs in a critical care setting.

## 5 Conclusion

From the perspective of health economics, the value of mNGS in diagnosing CNSIs is mainly reflected in the following aspects: First, improve the diagnostic efficiency: The application of mNGS diagnostic strategy can improve the cure rate of patients with CNSIs. The ability of mNGS to provide rapid diagnostic results helps to shorten the time to diagnose the disease, thus speeding up treatment decisions, which is of great significance for improving the cure rate of patients and reducing mortality. Second, reduce treatment costs: Accurate diagnosis results can help doctors choose more accurate treatment plans, avoid ineffective or unnecessary treatment, and thus reduce medical costs. For those cases of intracranial infection where the pathogen is difficult to identify using traditional methods, mNGS offers an effective solution. Third, promote personalized medicine: mNGS technology can provide more information about the pathogen, including its genetic variation, to help doctors develop customized treatment plans.

## Data Availability

The original contributions presented in the study are included in the article/**Supplementary Material**. Further inquiries can be directed to the corresponding authors.

## Supplementary material

Supplementary Figure 1 Linear regression of length of hospital stay and length of admission to ICU for all included patients.

Supplementary Figure 2 Linear regression between the cost of anti-infective therapy and total inpatient cost in mNGS and control groups. **(a)** Anti-infective treatment cost and total hospitalization cost in control group, *R2* = 0.5750, *P* < 0.0001; **(b)** The cost of anti-infective treatment and total hospitalization cost in the mNGS group, *R2* = 0.4832, *P* < 0.0001.
