# Supplementary figures and images for: Cost-effectiveness analysis of metagenomic next-generation sequencing versus traditional bacterial cultures for postoperative central nervous system infections in critical care settings: a prospective pilot study
Source: Front Cell Infect Microbiol. 2025 Oct 28;15:1710412. doi: 10.3389/fcimb.2025.1710412 (PMC12604022; doi:10.3389/fcimb.2025.1710412)

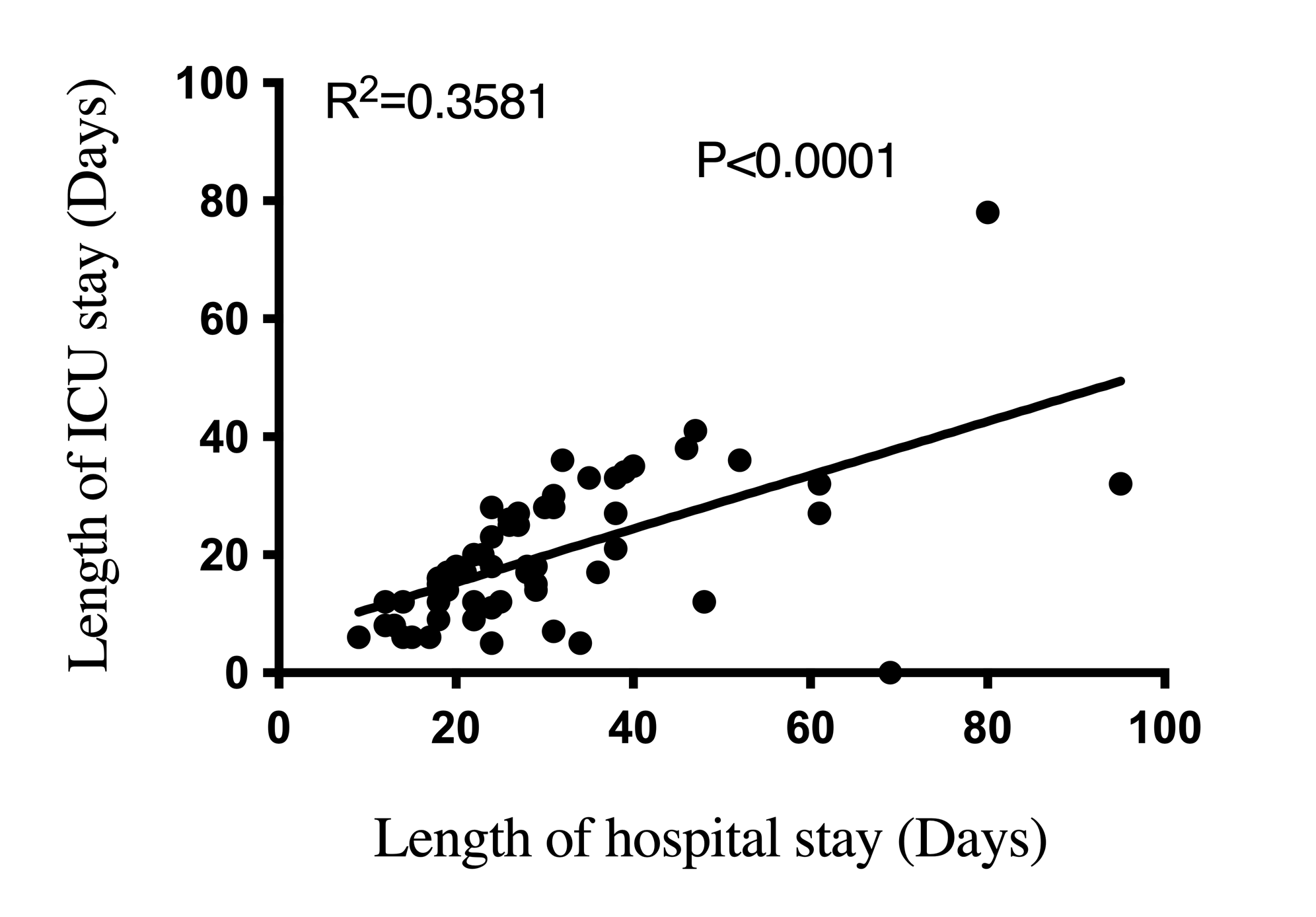

Supplement: Supplementary Figure 1 — Linear regression of length of hospital stay and length of admission to ICU for all included patients. [file Image1.tiff]

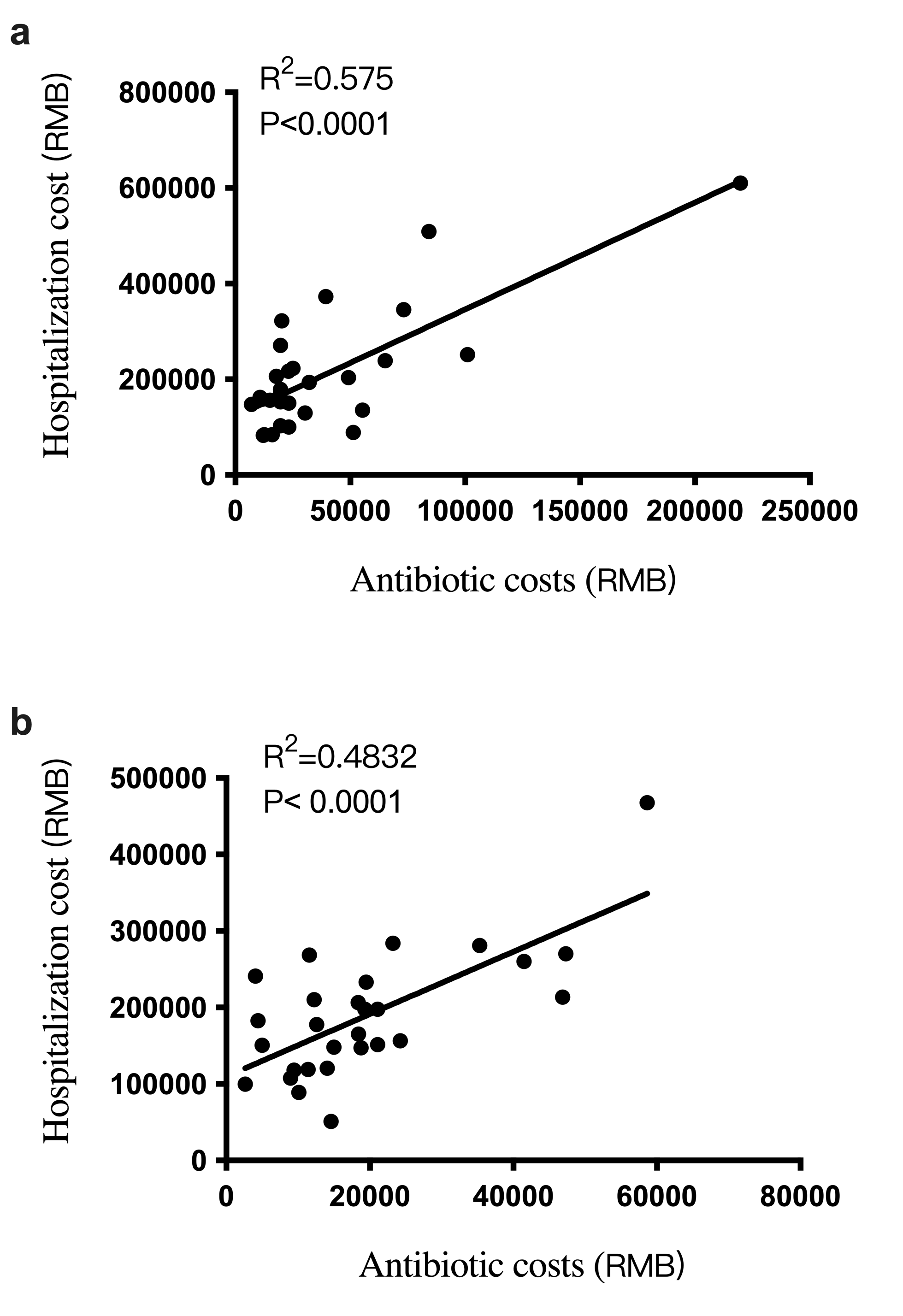

Supplement: Supplementary Figure 2 — Linear regression between the cost of anti-infective therapy and total inpatient cost in mNGS and control groups. (a) Anti-infective treatment cost and total hospitalization cost in control group, R2 = 0.5750, P < 0.0001; (b) The cost of anti-infective treatment and total hospitalization cost in the mNGS group, R2 = 0.4832, P < 0.0001. [file Image2.tiff]
